# A novel pathogenic splice acceptor site germline mutation in intron 14 of the APC gene in a Chinese family with familial adenomatous polyposis

**DOI:** 10.18632/oncotarget.15570

**Published:** 2017-02-20

**Authors:** Dan Wang, Shengyun Liang, Zhao Zhang, Guoru Zhao, Yuan Hu, Shengran Liang, Xipeng Zhang, Santasree Banerjee

**Affiliations:** ^1^ Department of Pathology, Tianjin Medical University General Hospital, Tianjin 300052, China; ^2^ Shenzhen Institutes of Advanced Technology, Chinese Academy of Sciences, Shenzhen 518055, China; ^3^ Department of Colorectal Surgery, Tianjin Union Medical Center, Tianjin 300121, China; ^4^ Department of Hematology, Third Affiliated Hospital, Sun Yat-Sen University, Guangzhou 510006, China; ^5^ School of Life Science and Biopharmaceuticals, Guangdong Pharmaceutical University, Guangzhou 510006, China; ^6^ Department of Cell Biology and Medical Genetics, School of Medicine, Zhejiang University, Hangzhou 310000, China

**Keywords:** familial adenomatous polyposis, APC gene, splice acceptor site mutation, targeted next-generation sequencing, colorectal cancer

## Abstract

Familial adenomatous polyposis (FAP) is an autosomal dominant precancerous condition, clinically characterized by the presence of multiple colorectal adenomas or polyps. Patients with FAP has a high risk of developing colorectal cancer (CRC) from these colorectal adenomatous polyps by the mean age of diagnosis at 40 years. Germline mutations of the *APC* gene cause familial adenomatous polyposis (FAP). Colectomy has recommended for the FAP patients with significant polyposis. Here, we present a clinical molecular study of a four generation Chinese family with FAP. Clinical diagnosis of FAP has been done according to the phenotype, family history and medical records. Patient's blood samples were collected and genomic DNA was extracted. In order to identify the pathogenic mutation underlying the disease phenotype targeted next-generation sequencing and confirmatory sanger sequencing has undertaken. Targeted next generation sequencing identified a novel heterozygous splice-acceptor site mutation [c.1744-1G>A] in intron 14 of *APC* gene, which is co-segregated with the FAP phenotypes in the proband and amongst all the affected family members. This mutation is not present in unaffected family members and in normal healthy controls of same ethnic origin. According to the LOVD database for Chinese colorectal cancer patients, in Chinese population, 60% of the previously reported *APC* gene mutations causes FAP, are missense mutations. This novel splice-acceptor site mutation causing FAP in this Chinese family expands the germline mutation spectrum of the APC gene in the Chinese population.

## INTRODUCTION

Familial adenomatous polyposis (FAP, OMIM #175100) is an autosomal dominant precancerous condition characterized by the appearance of hundreds to thousands of colorectal adenomas with an incidence of 3-10/100,000 [1, 2]. It is an autosomal dominant colon cancer predisposition syndrome accounting for approximately 1% of all colorectal cancers (CRC). Gradually, patients with FAP have high risk of developing colorectal cancer (CRC) by these colorectal adenomatous polyps if not treated early by total proctocolectomy [3, 4]. The risk of cancer is virtually 100% if the polyps are not detected and removed in time [5]. FAP has an almost equal worldwide distribution among males and females [6]. Usually, colorectal adenomatous polyps are begun to develop in the teenage years in classical FAP, if untreated, gradually increase the number of colorectal adenomatous polyps and causes CRC [7]. Patients with FAP may also develop extracolonic manifestations such as desmoids tumors, osteomas, dental abnormalities, congenital hypertrophy of the retinal pigment epithelium (CHRPE), lipomas, epidermoid cysts and upper gastrointestinal polyps along with colorectal adenomatous polyps [8]. In rare case, FAP is also associated with thyroid cancer, brain cancer and cancers of hepatobiliary tract. FAP is categorized into two types, namely; the classical FAP (CFAP) and the attenuated FAP (AFAP) on the basis of the number of colorectal adenomas or polyps and the age of onset of the patient [9]. Patients having CFAP are generally characterized by presence of more than 100 colorectal adenomatous polyps with an early age of onset for both polyposis and CRC. Patients with AFAP characteristically have 10–100 colorectal adenomatous polyps, with a late age of onset for both polyposis and CRC. [10].

Germline mutations of the *APC* gene cause FAP. *APC*, a tumor suppressor gene, comprises of 15 exons, encodes a large multi-domain protein composed of 2843 amino acid residues [11]. *APC* gene is expressed ubiquitously in various tissues, more specifically throughout the large intestine and central nervous system. *APC* is a multi-domain and complex functional protein. *APC* protein combines with glycogen synthesis kinase 3β (GSK-3β) and axin to form a complex called “destruction complex”, which prevents the accumulation of β-catenin in the cytoplasm [12]. Hence, mutation of the *APC* gene or inactivation of *APC* protein leads to the stimulation of the *Wnt* signaling pathway through decreased degradation of β-catenin which enables β-catenin to translocate into the nucleus, where it actives transcription factor TCF/LEF to increase the expression of several oncogenes, such as c-myc, cyclin D1, and MMP9 [13]. In addition, *APC* binds to cytoskeletal proteins including F-actin and microtubules to regulate cell migration and adhesion [5]. Moreover, *APC* also has a significant role in transcriptional activation, apoptosis, DNA repair, and meiosis [5, 14–17].

Pathogenic germline mutations of *APC* gene are classified into three major categories, i.e., nonsense/frameshift mutations, splice sites mutations and deep intronic deletions which results into large genomic rearrangements, followed by the formation of truncated *APC* proteins [18]. In few cases, point mutations or missense variants within the coding sequence of *APC* gene also result in the formation of alternative transcripts due to aberrant splicing [19]. For analyzing splice-site mutations, we found that the GT and AG sequence motifs are highly conserved consensus splice donor and acceptor site sequences at the intron-exon boundaries [20]. Splice site mutations accounting for ∼15% of pathogenic point mutations of various genes and the majority of splice site mutations are with splice donor (SD) site defects. However, it has been showed that the splice acceptor (SA) site mutations mostly involve the invariant AG sequence and finally results into exon skipping. In addition, approximately 13% of all SA mutations result in the creation of novel splice sites. Till date, only few SA mutations have been reported for *APC* gene. Previously published reports have shown that SA mutations either resulting in skipping of adjacent exon [21] or not functionally characterized SA substitutions, simply referred them as “splice acceptor defect” [22].

In HGMD, over 1100 different *APC* germline mutations have been reported in more than 3000 individuals till date (http://www.hgmd.cf.ac.uk/ac). In LOVD, 1198 different *APC* germline mutations have been reported in more than 3802 individuals till date (http://chromium.lovd.nl/LOVD2/colon_cancer/home.php?select_db=APC. In Chinese population, over 200 unique *APC* germline mutations have been reported from 275 individuals. (http://www.genomed.org/lovd2/home.php?select_db=APC). In addition, the 5′ end of the *APC* gene appears to be the hot-spots for germline mutations. Most interesting feature for pathogenic germline mutations of *APC* gene is that, the location of the mutation in the *APC* gene is very important as it is directly correlated with the phenotypic spectrum of the disease, age of onset and the appearance of extracolonic manifestations in FAP patients [3].

Here, in order to identify the genetic basis of polyposis in the affected relatives of this four generation Chinese family, we screened a panel of 14 genes (*APC, MLH1, MSH2, MSH6, PMS2, AXIN2, BMPR1A, EPCAM, MLH3, MUTYH, PMS1, PTEN, SMAD4, STK11*) associated with colorectal cancer by targeted exome capture based next-generation sequencing and confirmatory sanger sequencing. Here, we identified a germline heterozygous novel splice-acceptor site mutation in the invariant AG sequence of the intron 14 of the *APC* gene segregating with FAP phenotype among all the FAP patients in this four generation Chinese family, with autosomal dominant mode of inheritance.

## RESULTS

### Family recruitment and clinical examination

**Figure 1:**
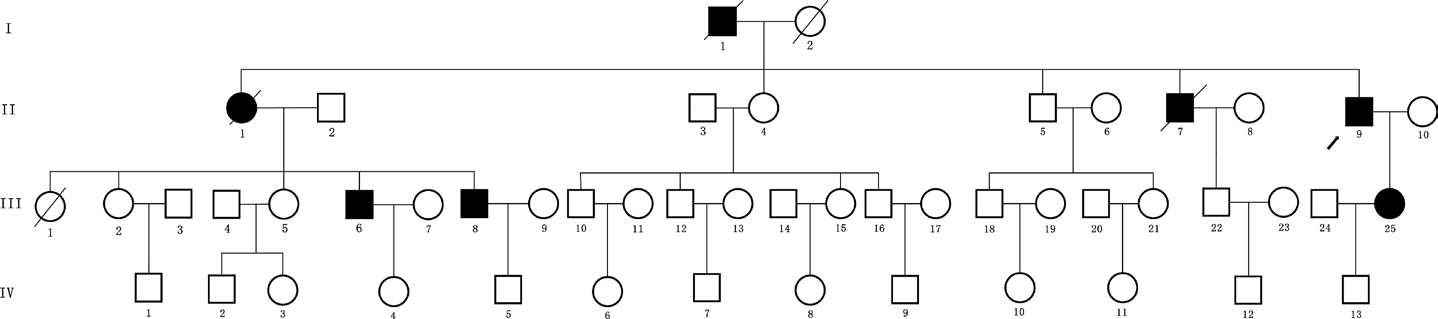
Pedigree of the Chinese family with familial adenomatous polyposis Family members with FAP are indicated with Shading. Squares and circles denoted males and females respectively. Individuals labeled with a solidus were deceased. Roman numerals indicate generations. Arrow indicates the proband (II-9).

**Figure 2:**
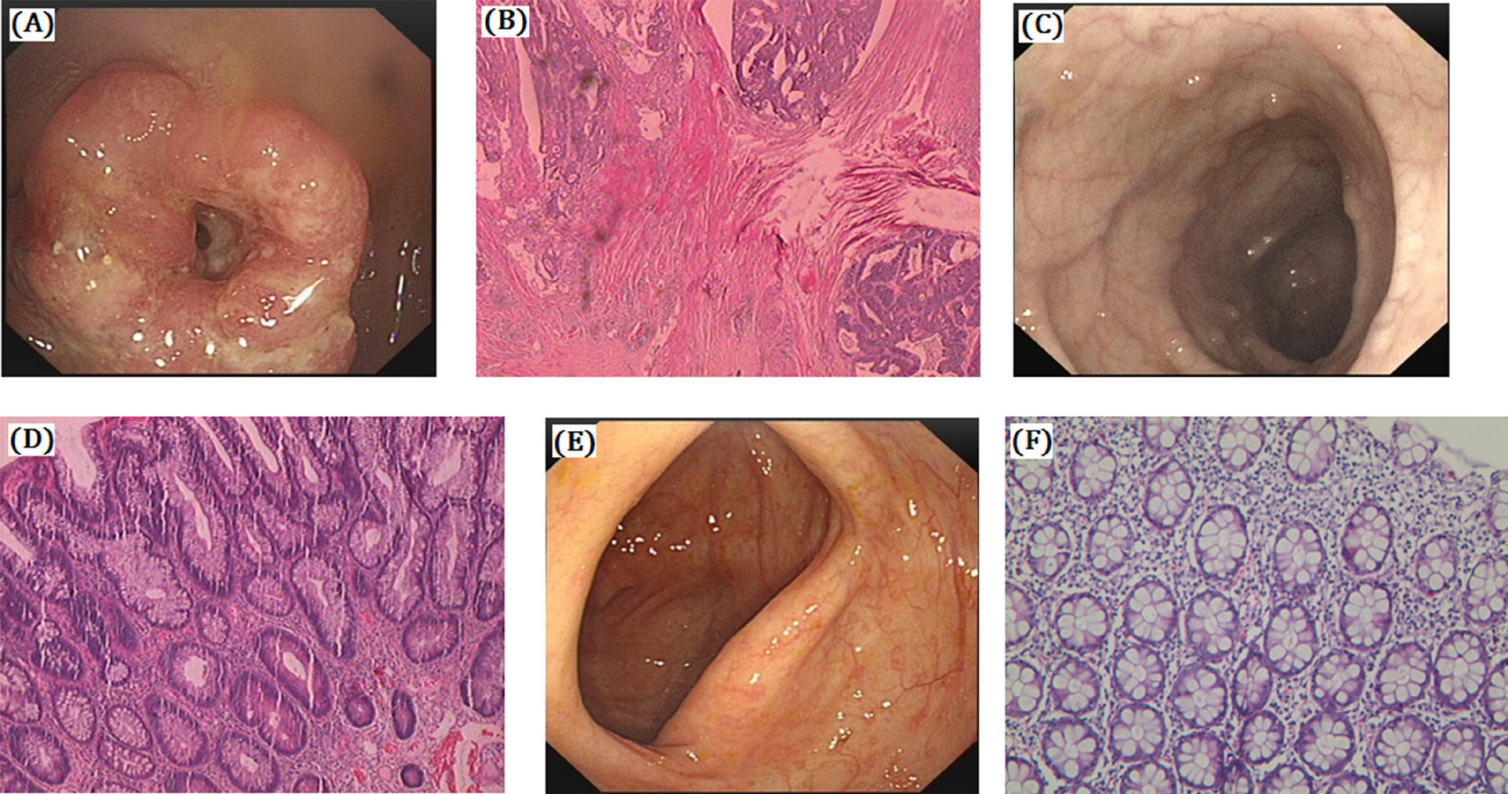
Clinical description (**A**–**B**) Colonoscopy and histology of the proband (II-9). Polyps of colon is High grade intraepithelial neoplasia (dysplasia), cancer invade the submucosa in proband (II-9). A. Protrude type mass in retucm, the mass take up the whole enteric cavity. (B) Moderately differentiated adenocarcinoma. (**C**–**D**) Colonoscopy and histology of the other affected members (III-25). Polyps of colon, cancer invade the submucosa (III-25). (C) Multiple polyps. (D) Tubular adenoma. (**E–F**) No polyps in the colon of unaffected member with normal histology (III-12). (E) Smooth intestinal tract. (F) Chronic inflammation of mucosa.

We identified a four generation Chinese pedigree with 45 members, among them four individuals were affected by FAP including three with CRC (Figure 1). There are 2 affected family members (I-1 and II-1) had died before from CRC. Another one affected family member (II-7) also died in his 70 years of age by Lung Cancer. The detailed description and comprehensive clinical information (present age, number of colorectal adenomas or polyps, clinical symptoms and extra-colonic manifestations) for all the family members are furnished in Table 1. Colonoscopy and histopathology of colon and rectum for the affected family members (II-9 and III-25) along with an unaffected member (III-12) are shown in Figure 2. The proband (II-9) has liver and lymph node metastasis. Diarrhea and hematochezia are seldom found in the proband (II-9). All the present (both affected and unaffected) family members are tested clinically for the disease.

**Table 1:** Clinical characteristics of all the affected and unaffected family members in this four generation Chinese family

Family ID	Sex	WT/MT	Present Age (Years)	No. of Colorectal Adenomas or Polyps	Clinical Symptoms	Extra-colonic Features
**I-1**	M		Died (80)	Unknown	Suspected FAP	
**I-2**	F		Died (82)		Died due to heart disease	
**II-1**	F		Died (79)	100–1000	Intestinal Cancer	
**II-2**	M	WT	75			
**II-3**	M	WT	85		-	
**II-4**	F	WT	80		-	
**II-5**	M	WT	75			
**II-6**	F	WT	70			
**II-7**	M		Died (70)	FAP(-)	Lung Cancer	
**II-8**	F	WT	65			
**II-9**	M	MT	61	100–1000	FAP/CRC	
**II-10**	F	WT	59			
**III-1**	F		Died (40)		Died due to heart disease	
**III-2**	F	WT	53		-	
**III-3**	M	WT	55		-	
**III-4**	M	WT	54			
**III-5**	F	WT	53			
**III-6**	M	MT	50	100–1000	FAP/CRC	
**III-7**	F	WT	48			
**III-8**	M	MT	42	100–1000	FAP/CRC	
**III-9**	F	WT	40			
**III-10**	M	WT	49			
**III-11**	F	WT	48			
**III-12**	M	WT	47			
**III-13**	F	WT	46			
**III-14**	M	WT	47		-	
**III-15**	F	WT	46		-	
**III-16**	M	WT	42		-	
**III-17**	F	WT	40		-	
**III-18**	M	WT	48		-	
**III-19**	F	WT	47		-	
**III-20**	M	WT	46		-	
**III-21**	F	WT	46			
**III-22**	M	WT	45			
**III-23**	F	WT	43			
**III-24**	M	WT	32			
**III-25**	F	MT	30	100–1000	FAP	
**IV-1**	M	WT	34			
**IV-2**	M	WT	30			
**IV-3**	F	WT	26			
**IV-4**	F	WT	26			
**IV-5**	M	WT	24			
**IV-6**	F	WT	24			
**IV-7**	M	WT	23			
**IV-8**	F	WT	20			
**IV-9**	M	WT	18			
**IV-10**	F	WT	18			
**IV-11**	F	WT	20			
**IV-12**	M	WT	20			
**IV-13**	M	WT	5			

### Clinical description

Proband (II-9) is a 61 years old male. The proband has a history of having intermittent diarrhea since last ten years. Proband also having a history of hematochezia for last three months. After physical examination of the proband we find there are tumors in his rectum. In order to diagnose clinically, colonoscopy has undertaken. Colonoscopy result showed that there are more than 100 polyps in colon and rectum. Protruding type of mass are identified in his rectum and the mass take up the whole enteric cavity. According to the clinical diagnosis, whole colorectal resection or total proctocolectomy was recommended. Postoperative pathology was showing moderately differentiated adenocarcinoma, mucinous adenocarcinoma at local, mesenteric lymph nodes followed by chemotherapy for 16 times (FOLFIRI). After two years of total proctocolectomy, it was observed that the liver and lung had metastasis and the proband had died after one year.

Another affected family member (III-6) is a male of 50 years of age. He also has a history of intermittent diarrhea for 8 years along with hematochezia for last two months. Colonoscopy showed canceration of rectal polyps. Total proctocolectomy was carried out. An affected family member (III-8) is a male of 42 years old. He also has a history of having intermittent diarrhea but hematochezia has not observed. Canceration of colorectal polyps was identified at early stage without metastasis. Lastly, another affected family member (III-25) is a female of 30 years old. She has a history of having intermittent diarrhea since 5 years without hematochezia. Colonoscopy showed 100-1000 colorectal polyps with tubular adenoma.

### Identification and characterization of novel splice-site mutation

**Figure 3:**
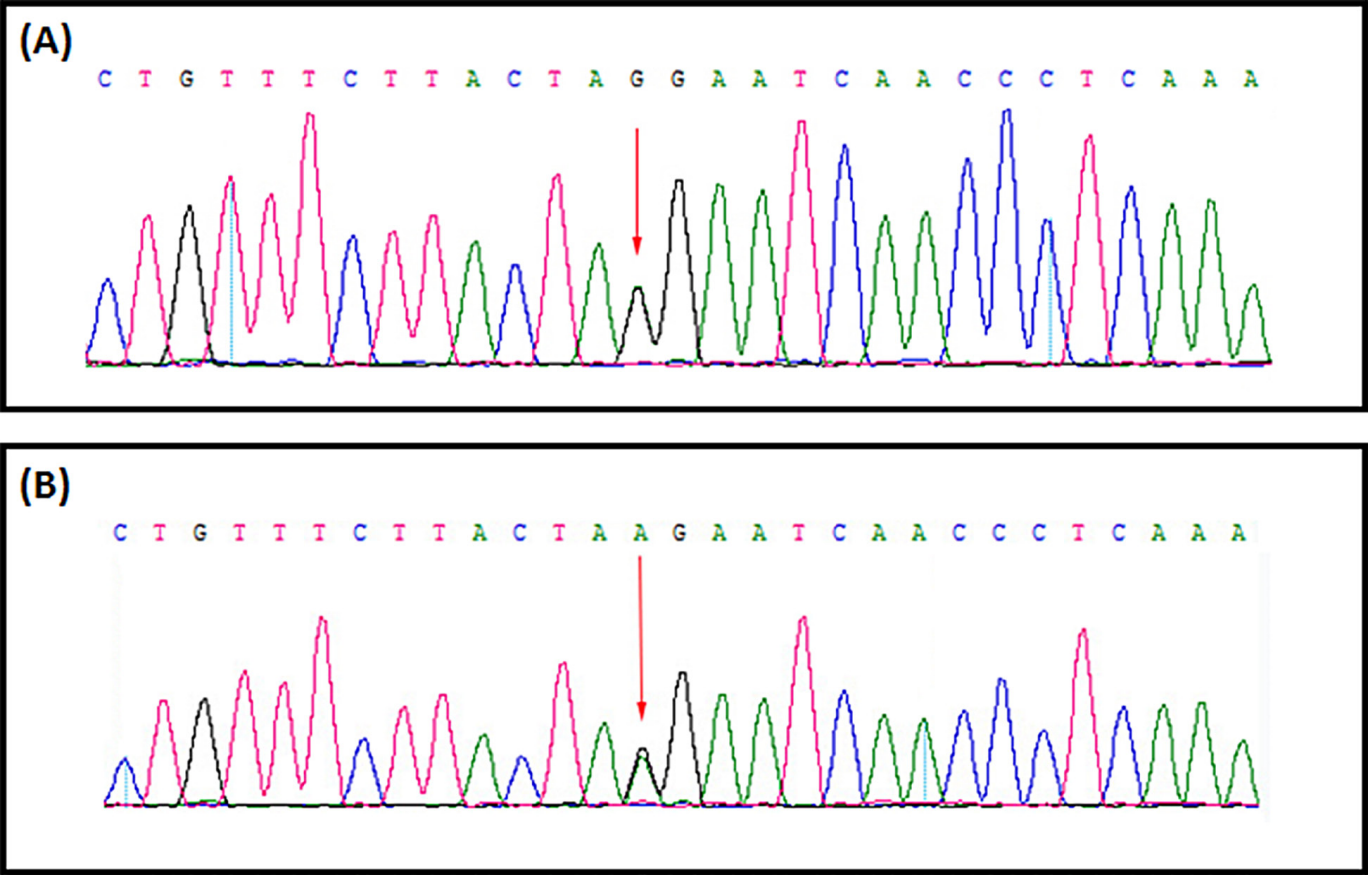
Sanger sequencing of the APC gene identified a germline novel heterozygous splice acceptor site mutation (GenBank Accession: NM_000038). (**A**) DNA sequence derived from unaffected family member (III-12). (**B**) The equivalent region from proband (II: 9) arrow showing the novel heterozygous splice acceptor site substitution mutation (c.1744-1G>A).

A heterozygous novel splice-site mutation; [c.1744-1G>A] in the intron 14 of the *APC* gene [NCBI Reference sequence NM_000038] was identified in proband (II-9) by targeted next generation sequencing (Figure 3). This heterozygous novel splice-site mutation is co-segregated with the FAP phenotypes in the proband (II-9) and amongst all the affected family members (III-6, III-8 and III-25) but absent in the unaffected family members. This splice-site mutation is also absent in the normal control of the same ethnic origin, gender and age range. All the present (both affected and unaffected) family members are tested for this splice-acceptor site mutation [c.1744-1G>A] of *APC* gene by Sanger sequencing.

### Bioinformatics analysis

#### *In silico* analysis

Human splicing finder (http://www.umd.be/HSF3/) [23] predicted that this heterozygous novel splice-acceptor site mutation causes loss of wild type splice acceptor site which most probably affecting the normal *APC* mRNA splicing event and leads to formation of alternative transcripts by aberrant splicing. Simultaneously, it has also predicted that this SA mutation also causes activation of new acceptor site or an intronic cryptic acceptor site which also finally results into formation of alternative transcripts.

## DISCUSSION

**Figure 4:**
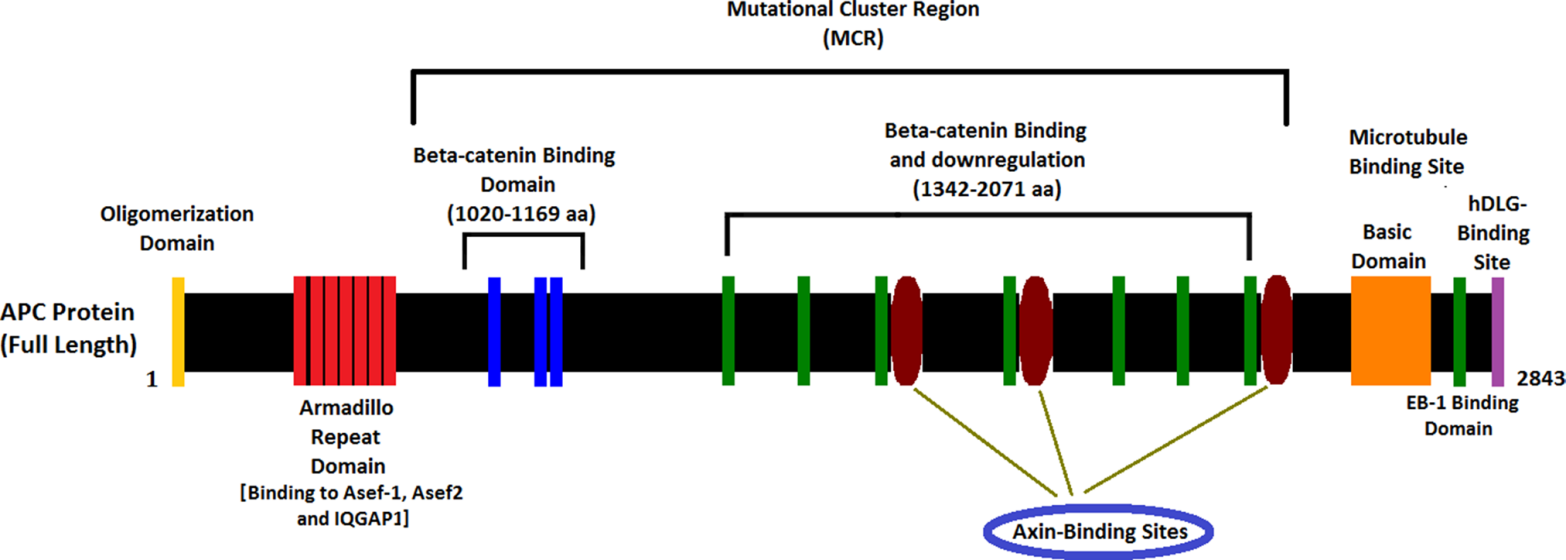
Schematic diagram of the APC protein structure with functional domains

In present study, we found a germline heterozygous novel splice-acceptor site mutation (c.1744-1G>A) in the canonical splice acceptor site on intron 14 [NCBI Reference sequence NM_000038] of the human *APC* gene in the proband (II-9) and amongst all the FAP-affected family members [III-6, III-8 and III-25] in a four generation Chinese family. This heterozygous novel splice-site mutation of *APC* gene probably affects the normal mRNA splicing events and results in the formation of alternative transcripts of *APC* protein by aberrant splicing. *APC* protein comprises of the armadillo repeat domain, β-catenin binding site, microtubule binding site, EBI Domain, hDLG binding site and PDZ binding domain (Figure 4). This novel heterozygous splice-acceptor site mutation of *APC* gene is classified as “likely pathogenic” according to the variant interpretation guidelines by American College of Medical Genetics and Genomics (ACMG). This mutation is not present in the ExAC database.

In this four generation Chinese family, clinical diagnosis of affected family members with FAP has been done on the basis of the colonoscopy report, review of clinical report, endoscopic report, histo-pathological data, and family history. Moreover, the autosomal dominant mode of inheritance of disease phenotype with the presence of FAP-associated clinical symptoms (colorectal adenomatous polyps) allows us to diagnose the disease in this family as FAP. In our present study, the heterozygous novel splice-site mutation of *APC* gene in this four generation Chinese family manifests with colorectal adenomatous polyps without any extra-colonic manifestations.

### Splice site mutation of APC gene

Splice-acceptor site mutations of *APC* gene are usually predicted to result in an exonic deletion and therefore further investigations of the mRNA transcripts are not always carried out [22]. There are two possible hypotheses regarding the effect of this mutation in *APC* protein. Firstly, if this SA (spilce-acceptor site) substitution in this family were to cause exon skipping, then it would have resulted in an *in frame* deletion of *APC* exon 15. Such a mutant *APC* transcript would have a partial loss of the axin binding sites of β-catenin binding and down-regulation domain belongs to mutation cluster region (MCR). Secondly, the creation of new cryptic splice acceptor site in the exon 15 resulting in the formation of truncated *APC* exon 15. Loss of entire exon 15 or truncated exon 15 leads to loss of β-catenin binding domain of *APC* has been associated with the classical FAP phenotype [7]. In this Chinese family, all affected patients have presented with the classical polyposis phenotype, supporting either of this hypothesis.

### Splice-acceptor site mutation in exon 15 of APC gene

Exon 15 of *APC* gene is the most common and major target for both germline and somatic mutations with >75% of the coding sequence of *APC* [24]. It has been reported before that the germline mutations in the 3′ part of *APC* exon 15 do not result in truncated proteins and the 5′ half of exon 15 of the *APC* gene harboring the majority of the germline mutations [25–28]. *APC* germline mutations in exon 15 were found in 61% patients, whereas APC somatic mutations were localized to 22% patients with duodenal adenomas [29].

### Mutations at intron 14-Exon 15 boundary of Exon15 of APC gene

Aretz S et al., reported that the substitution (c.1744G>A) of the first position of exon 15 results into skipping of exon 15 in FAP patients [19]. But later Plawski A et al., reported that the substitution of the first base from G to T (c.1744G>T; p.Glu582X) in the exon15 of *APC* gene causes creation of a premature stop codon which leads to the formation of a truncated exon15 of *APC* gene [30]. According to the previous report c.1744-2A>G also results into skipping of full exon15 from the *APC* gene [19, 22]. According to an unpublished data from *APC* mutation database of “The Universal Mutation Database” (UMD; http://www.umd.be/APC/), c.1744-1G>C is enlisted in the FAP patient and it was predicted to break the wild type splice-acceptor site which leads to the formation of alternative transcripts by aberrant splicing.

Hence, in this present case, c.1744-1G>A in intron 14 of *APC* gene is most possibly causes disease phenotype in FAP patients by breaking the wild type splice-acceptor site at intron14-exon15 boundary of *APC* gene which leads to the formation of alternative transcripts by aberrant splicing.

In conclusion, the present study describes a germline novel heterozygous splice-acceptor site mutation in *APC* gene in a four generation Chinese family with FAP. Our study expands the genotype-phenotype correlation along with spectrum of the germline mutations of *APC* gene in the Chinese population. Our novel finding could contribute to a more comprehensive database of germline mutations of *APC* gene that could be used for the early molecular diagnosis, risk assessment, susceptibility of the disease for the FAP patients. Improved understanding of both the genetics and patho-biology of *APC* may, in time, culminate in preventative or therapeutic strategies specifically targeted at reducing the burden of colorectal cancer.

## MATERIALS AND METHODS

### Ethical statement

Family members of this four generation Chinese family have given written informed consent as they are participating in this study. The Ethical Committee of the Tianjin Union Medical Center, China, reviewed and approved our study protocol in compliance with the Helsinki declaration. Diagnosis of the patients for FAP has done by oncologists, on the basis of clinical test reports and detailed family pedigree.

### Patients and pedigree

A four generation Chinese family with FAP (Figure 1), diagnosed and treated in the Department of Colorectal Surgery, Tianjin Union Medical Center, 300121, China, were enrolled in our study. Clinical diagnosis of FAP was established in this family by endoscopic screening after the proband (III-1) presented to Tianjin Union Medical Center with CRC. The diagnostic standard or criteria for patients with FAP was as follows: (1) patients having >100 colorectal adenomas or polyps and (2) at least 20 synchronous colorectal adenomas or polyps in patients with a positive family history of FAP.

### Targeted exome-based next-generation sequencing and variant identification

DNA samples obtained from the proband (II-9) were sequenced using target exome-based next-generation sequencing. Roche NimbleGen's (Madison,USA) custom Sequence Capture Human Array was used to designed to capture 98480 kb of targeted sequence, covering 181 exons and flanking sequence (including the 100 bp of introns) of 14 genes (*APC, MLH1, MSH2, MSH6, PMS2, AXIN2, BMPR1A, EPCAM, MLH3, MUTYH, PMS1, PTEN, SMAD4, STK11*) which is associated colorectal cancer (CRC) and yielded an average of 6366534 reads per sample, with approximately 68.78% mapping to the targeted regions. The average sequencing depth of the target area is 303.39% with 99.51% coverage. The procedure for preparation of libraries was consistent with standard operating protocols. In each pooling batch, 10 to 33 samples were sequenced simultaneously on Illumina HiSeq 2500 Analyzers (Illumina, San Diego, USA) for 90 cycles (specially designed by us for this study). Image analysis, error estimation, and base calling were performed using Illumina Pipeline software (version 1.3.4) to generate raw data. The raw reads were screened to generate – clean reads‖ followed by established filtering criteria. Clean reads with a length of 90 bp were aligned to the reference human genome from the NCBI database (Build 37) using the Burrows Wheeler Aligner (BWA) Multi-Vision software package with output files in - bam‖ format. The bamdata were used for reads coverage in the target region and sequencing depth computation, SNP and INDEL calling, and CNV detection. First, a novel three-step computational frame work for CNV was applied. Then, SNPs and INDELs were called using SOAPsnp software and Sam tools pileup software, respectively. A SNP or INDEL was be filtered if it could not follow the criterion: supported by at least 10 reads and > 20% of the total reads. The frequency filter was set at 0.05. If a SNP frequency was more than 0.05 in any of the four databases (dbSNP, Hapmap, 1000 Genomes Project, the 124 healthy reference samples sequenced in this study), it would be regarded as a polymorphism, but not a causative mutation.

Last, SNVs were retrieved in The Human Gene Mutation Database (http://www.hgmd.cf.ac.uk/ac/index.php) and the Leiden Open Variation Database (http://www.lovd.nl/3.0/home), and then labeled as reported or novel.

### Confirmation of the novel splice-site mutation by sanger sequence

To validate true positive of the mutation, Sanger sequencing was performed. Primers flanking the candidate loci were designed based on the reference genomic sequences of Human Genome from GenBank in NCBI and synthesized by Invitrogen, Shanghai, China. PCR amplification was carried out in ABI 9700 Thermal Cycler. PCR products were directly sequenced on ABI PRISM 3730 automated sequencer (Applied Biosystems, Foster City, CA, USA). Sequence data comparisons and analysis were performed by DNASTAR SeqMan (DNASTAR, Madison, Wisconsin, USA).

The heterozygous novel splice-acceptor site mutations identified through targeted next generation sequencing were verified through Sanger sequencing using the primers: F1 5′-TTTACCAGTGAGGGACGGGC-3′, R1 5′-GTTTGTCTGGCTCCGGTAAGTA-3′. The reference sequence NM_000038 of APC was used.

### Bioinformatics analysis

#### *In silico* analysis

This heterozygous novel splice site mutation is located at splice-acceptor site between intron 14 and exon 15. In order to understand the effect of this mutation on *APC* mRNA splicing, we have done *in silico* analysis.

In our *in silico* analysis we identified the effect of this mutation on APC mRNA splicing by Human splicing finder (http://www.umd.be/HSF3/) [23].
